# ECoG high gamma activity reveals distinct cortical representations of lyrics passages, harmonic and timbre-related changes in a rock song

**DOI:** 10.3389/fnhum.2014.00798

**Published:** 2014-10-13

**Authors:** Irene Sturm, Benjamin Blankertz, Cristhian Potes, Gerwin Schalk, Gabriel Curio

**Affiliations:** ^1^Berlin School of Mind and Brain, Humboldt Universität zu BerlinBerlin, Germany; ^2^Neurotechnology Group, Department of Electrical Engineering and Computer Science, Berlin Institute of TechnologyBerlin, Germany; ^3^Neurophysics Group, Department of Neurology and Clinical Neurophysiology, Charité — University Medicine BerlinBerlin, Germany; ^4^Bernstein Focus: NeurotechnologyBerlin, Germany; ^5^National Resource Center for Adaptive Neurotechnologies, Wadsworth Center, New York State Department of HealthAlbany, NY, USA; ^6^Department of Electrical and Computer Engineering, University of Texas at El PasoEl Paso, TX, USA; ^7^Department of Neurosurgery, Washington University in St. LouisSt. Louis, MO, USA; ^8^Department of Biomedical Engineering, Rensselaer Polytechnic InstituteTroy, NY, USA; ^9^Department of Neurology, Albany Medical CollegeAlbany, NY, USA; ^10^Department of Neurosurgery, Washington University in St. LouisSt. Louis, MO, USA

**Keywords:** music processing, natural music, electrocorticography (ECoG), high gamma, acoustic features

## Abstract

Listening to music moves our minds and moods, stirring interest in its neural underpinnings. A multitude of compositional features drives the appeal of natural music. How such original music, where a composer's opus is not manipulated for experimental purposes, engages a listener's brain has not been studied until recently. Here, we report an in-depth analysis of two electrocorticographic (ECoG) data sets obtained over the left hemisphere in ten patients during presentation of either a rock song or a read-out narrative. First, the time courses of five acoustic features (intensity, presence/absence of vocals with lyrics, spectral centroid, harmonic change, and pulse clarity) were extracted from the audio tracks and found to be correlated with each other to varying degrees. In a second step, we uncovered the specific impact of each musical feature on ECoG high-gamma power (70–170 Hz) by calculating partial correlations to remove the influence of the other four features. In the music condition, the onset and offset of vocal lyrics in ongoing instrumental music was consistently identified within the group as the dominant driver for ECoG high-gamma power changes over temporal auditory areas, while concurrently subject-individual activation spots were identified for sound intensity, timbral, and harmonic features. The distinct cortical activations to vocal speech-related content embedded in instrumental music directly demonstrate that song integrated in instrumental music represents a distinct dimension in complex music. In contrast, in the speech condition, the full sound envelope was reflected in the high gamma response rather than the onset or offset of the vocal lyrics. This demonstrates how the contributions of stimulus features that modulate the brain response differ across the two examples of a full-length natural stimulus, which suggests a context-dependent feature selection in the processing of complex auditory stimuli.

## 1. Introduction

The appreciation for music is a universal human capacity that plays an inspiring role in our individual and social lives. Music is processed in a cascade of steps that lead from the segregation within the auditory stream, the extraction and integration of a variety of acoustic features, to cognitive, memory-related processes that induce personal, often emotional, experiences. Critical structural components of music have been analyzed in studies addressing the processing of pitch (Hyde et al., 2008; Kumar et al., 2011; Nan and Friederici, 2013; Plack et al., 2014), sensory dissonance (Regnault et al., 2001; Perani et al., 2010; Daikoku et al., 2012), timbre (Deike et al., 2004; Goydke et al., 2004; Caclin et al., 2006, 2007), melodic contour (Trainor et al., 2002), key (Janata et al., 2002), mode (Halpern et al., 2008), scale properties (Brattico et al., 2006), music-syntactic congruity (Koelsch et al., 2002; Sammler et al., 2011, 2013; Jentschke et al., 2014; Kim et al., 2014) and rhythmic aspects (Jongsma et al., 2004; Snyder and Large, 2005; Grahn and Rowe, 2009; Abrams et al., 2011; Schaefer et al., 2011a). Typically, these approaches rely on carefully selected or specifically designed stimulus material that allows to examine one aspect of music in an isolated manner while controlling for other influences. This approach has provided a large corpus of evidence about associations between specific aspects of music and brain areas. Notably, by design, it does not directly address the confluence of the multitude of musical features and their intrinsic relations. Including this integrative aspect of musical compositions could contribute to a comprehensive and veridical picture of brain responses to music. Brain responses to naturalistic stimulation may differ from those related to controlled stimulation with simplified stimuli, as suggested by evidence from both the visual and the auditory domain (Hasson et al., 2010 or Abrams et al., 2013, respectively). Abrams et al. provided (to our knowledge) the first direct evidence that the between-subject synchronization of a large-scale distributed network including auditory midbrain and thalamus, auditory cortex, parts of frontal and parietal cortex, and motor planning regions was significantly higher when listeners were presented with complex musical stimuli lasting minutes than when they listened to shorter pseudo-musical contexts.

Accordingly, a stimulus should be sufficiently long to represent a complex musical context, and the music material should be naturalistic and free of manipulations to approximate ecological validity.

These requirements imply a complex, often unbalanced, stimulus material and the single presentation of one (long) stimulus without a-priori defined chances for repetition and signal averaging. In spite of this challenge for data analysis, the interest in the processing of natural music has recently grown considerably. A number of studies using naturalistic music stimuli examine relations between brain signals and behavioral measures, such as autobiographic salience (Janata, 2009), expressive performance (Chapin et al., 2010), emotion ratings (Mikutta et al., 2012, 2013), or ratings of perceived tension (Lehne et al., 2014). Several approaches that combine neuroimaging and acoustic feature extraction directly investigate the relation between brain signals and the multi-dimensional structure of music (Alluri et al., 2012, 2013; Toiviainen et al., 2014) or investigate the inter-subject synchronization of brain responses to naturalistic music (Abrams et al., 2013; Potes et al., 2014). Only few of these studies used the electroencephalogram (EEG), which due to its high temporal resolution is suitable for investigating the dynamics of music on a fine-grained time scale but typically relies on averaging. Apart from (Mikutta et al., 2012, 2013) where EEG recordings are related to behavioral measures, one novel approach to analyze ongoing EEG elicited by natural music stimuli has been proposed in Cong et al. (2012). It allows to identify EEG components that are common to the majority of subjects and, subsequently, compares the time course of these components to music features. Common to all approaches mentioned above is that they are only sensitive to effects that occur with a certain degree of (spatial) consistency within the group of subjects.

Electrocorticographic recordings (ECoG) from the brain surface provide additional benefits since their superior signal-to-noise ratio is advantageous for the analysis of single stimulus presentations at the level of single subjects. They combine high temporal resolution with high spatial resolution. Thus, they offer a much higher level of spatial specificity and an extended frequency range compared to scalp-recorded EEG. In the field of speech perception research, ECoG has emerged as a new technique to study the functional cortical organization of speech processing (Pasley et al., 2012; Kubanek et al., 2013; Leonard and Chang, 2014; Martin et al., 2014) while studies on music perception are still rare. A first example how the time course of sound intensity of a naturalistic music stimulus can be tracked in ECoG features was provided by Potes et al. (2012). Specifically, this study revealed that high-gamma band (70–170 Hz) ECoG activity in the superior temporal gyrus as well as on the dorsal precentral gyrus is highly correlated with the time course of sound intensity in a continuous stream of natural music. A subsequent study by Kubanek et al. (2013) found that high-gamma ECoG activity also tracks the temporal envelope of speech and compared it to the activations related to music, identifying different levels of specificity in an auditory network constituted by the auditory belt areas, the superior temporal gyrus (STG) and Broca's area. Very recently, a new analysis of the same data set identified spatial and causal relationships between alpha and gamma ECoG activity related to the processing of sound intensity (Potes et al., 2014). Considering that sound intensity (a technical proxy for perceived loudness) was tracked in ECoG features with significant robustness, the same data set appears highly promising for a further investigation that takes into account the variety of features available in this natural music stimulus, a rock song.

The goal of the present follow-up analysis was to explore whether music-related variables other than sound intensity can be tracked in ECoG and, if so, how respective areas of cortical activation compare to those associated with the processing of sound intensity in Potes et al. (2012). Because a naturalistic music stimulus contains different perceptual dimensions that are intrinsically related, it was a critical challenge to differentiate these in the brain response. In addition to the feature of sound intensity that was investigated in the previous studies, we chose four features that relate to different aspects of music. These include the moment-to-moment distinction vocals on/off, a continuous measure of harmonic change probability, a measure related to timbral aspects (spectral centroid), and a rhythm-related measure (pulse clarity) (for details see Materials and Methods).

## 2. Materials and methods

### 2.1. Subjects and data collection

We analyzed data from ten subjects (for patient's clinical profiles see Table 1 in the Supplemental Data). These 10 subjects included seven of the eight subjects who were analyzed in our previous study (Potes et al., 2012) where patients with epilepsy (4 women, 4 men) were instructed to listen attentively (without any other task) to a single presentation of the rock song “Another Brick in the Wall - Part 1” (Pink Floyd, Columbia Records, 1979) while ECoG activity was recorded. We added to this dataset data from three additional subjects who followed the same protocol. In all patients in the present analysis the electrode grid was in the left hemisphere. All subjects gave informed consent to participate in the study, which was approved by the Institutional Review Board of Albany Medical College. None of the subjects had a history of hearing impairment. The total numbers of implanted electrodes were 96, 83, 109, 58, 120, 58, 59, 112, 134, and 98 for subjects S1 to S10, respectively. After removal of channels containing environmental or other artifacts, 86, 82, 104, 56, 108, 57, 53, 93,110, and 92 channels were left for analysis. Grid placement and duration of ECoG monitoring were based solely on the requirements of the clinical evaluation without any consideration of this study. Each subject had postoperative anterior–posterior and lateral radiographs, as well as computer tomography (CT) scans to verify grid locations. The song was 3:10 min long, digitized at 44.1 kHz in waveform audio file format, and binaurally presented to each subject using in-ear monitoring earphones (12–23.5 kHz audio bandwidth, 20 dB isolation from environmental noise). ECoG signals were referenced to an electrocorticographically silent electrode (i.e., a location that was not identified as eloquent cortex by electrocortical stimulation mapping), digitized at 1200 Hz, synchronized with stimulus presentation, and stored with BCI2000 (Schalk et al., 2004; Schalk and Mellinger, 2010). In addition, we analyzed data from the same subjects where they listened to the presentation of four narrated stories that are part of the Boston Aphasia Battery (Goodglass et al., 1983) (details see Kubanek et al., 2013).

### 2.2. Extraction of ECoG features

Our analysis focused on the high-gamma band. ECoG activity in the high gamma band has generally been associated with functional activation of the cortex in different domains (Crone et al., 2006). For auditory and speech perception, numerous studies have shown that ECoG high-gamma power modulations over auditory areas provide important information about the spatio-temporal dynamics of sound processing (Edwards et al., 2005; Towle et al., 2008; Pei et al., 2011; Pasley et al., 2012; Potes et al., 2014). We extracted ECoG high-gamma power using the same method as in Potes et al. (2012): high-gamma (70–170 Hz) amplitudes were extracted by first applying a 0.1 Hz high-pass filter and then a common average reference (CAR) spatial filter to the ECoG signals. For every 50 ms window, we estimated a power spectrum from the time-series ECoG signal using an autoregressive (AR) model. Spectral magnitudes were averaged for all frequency bins between 70 and 115 and between 130 and 170 Hz (omitting line noise at 120 Hz).

### 2.3. Selection of music features

From the large number of potential features that characterize a music audio signal, we chose a set of five features that capture salient dynamic features of the stimulus and cover a broad spectrum of structural categories of music. Since the results of Potes et al. (2012) revealed a strong correlation of ECoG high-gamma power fluctuations with the sound intensity of the continuous music stimulus, sound intensity was chosen as first feature. It is a temporal feature that can be extracted directly from the raw audio signal and can be considered as an approximate measure of loudness. The second feature was the logarithmic spectral centroid, which is perceptually related to the complex property of timbre. More specifically, it has been related to perceived brightness of sound in Schubert et al. (2004) and to perceived pitch level in Coutinho and Cangelosi (2011). The third feature was probability of harmonic change, which relates to higher-level musical structure, i.e., to harmonic progression and musical syntax. Pulse clarity as fourth feature indicates how easily listeners perceive the underlying rhythmic or metrical pulsation of a piece of music. This feature has been introduced and perceptually validated in Lartillot et al. (2008b) and since then has been used in numerous studies (Eerola et al., 2009; Zentner, 2010; Higuchi et al., 2011; Alluri et al., 2012; Burger et al., 2013). Since an essential characteristic of the music stimulus is the presence of song (lyrics), the fifth feature, vocals on/off, captures the change between purely instrumental passages and passages with vocal lyrics content.

In summary, we chose a description of the audio signal that relates to important basic variables of the perception of music: loudness, timbre, and rhythm. With harmonic change, it encompasses also an abstract high-level property related to the rules of Western major-minor harmony. Finally, with vocals on/off, it allows also to address the impact of vocals with lyrics in music. For comparison, in a complementary analysis, the identical analysis was applied to the sound files of the speech stimuli.

### 2.4. Extraction of music features

Sound intensity was calculated in Matlab (The MathWorks Inc., Natick, Massachusetts). Vocals on/off was determined manually. All other features were extracted using freely available software (see below). We used the first 125 s of Pink Floyd's The Wall - part 1 in the analysis since the last minute of the song is an instrumental afterlude passage with considerably less variation, in particular without any vocal parts.The five features were calculated as described in the following sections.

#### 2.4.1. Sound intensity

The sound intensity of the audio signal was calculated as the average power derived from 50 ms segments of the audio waveform overlapping by 50%. The resulting time course was downsampled to match the sampling rate of 20 Hz of the extracted ECoG high gamma power.

#### 2.4.2. Vocals on/off

The presence of vocals was annotated manually in the audio file. This annotation resulted in a binary function that contained the value 1 for passages with lyrics and 0 otherwise. In the music stimulus there are seven passages with vocal lyrics with average duration of 4.22 s (±0.77) that are separated by at least 5 s of purely instrumental music. In a complementary analysis, we applied a similar procedure to the speech stimuli. Here, 0 was assigned to passages of silence within the story that exceeded the duration of 400 ms, such as pauses between sentences or phrases, while 1 denoted ongoing speech. In the speech stimulus the duration of speech passages was shorter (mean duration 1.65 s ±0.55) and vocals on/off changes occurred more frequently (30 changes in 100 s). In both stimuli the analyzed data start with the first tone of the song or with the first sentence of the narration, respectively, not including a silent pre-stimulus period.

#### 2.4.3. Spectral centroid

The centroid of the log-scaled frequency spectrum was calculated for 50% overlapping windows of 50 ms using the implementation in the MIRtoolbox (Lartillot et al., 2008b). The spectral centroid is the amplitude-weighted mean frequency in a window of 50 ms. It is an acoustic measure that indicates where the “mass” of the spectrum is located. The log-scaled centroid was downsampled to match the sampling rate of 20 Hz of the extracted ECoG high gamma power.

#### 2.4.4. Pulse clarity

Pulse clarity was calculated for windows of 3 s with a 33% overlap using the MIRtoolbox (Lartillot et al., 2008b), then interpolated to match the ECoG sampling frequency of 20 Hz. Pulse clarity is a measure of how strong rhythmic pulses and their periodicities can be perceived by the listener. It is based on the relative Shannon entropy of the fluctuation spectrum (Pampalk et al., 2002) and has been perceptually validated as being strongly related to listener's perception of the degree of rhythmicity in a piece of music in Lartillot et al. (2008a).

#### 2.4.5. Harmonic change

The harmonic change function measures the probability of a harmonic change and detects chord changes. We derived this metric using the Queen Mary plugin for the sonic visualizer (del Bimbo et al., 2010), which implements an algorithm that was proposed and validated on a selection of rock songs in Harte et al. (2006). The algorithm comprises a segmentation of the audio signal into 50 ms windows, spectral decomposition of each window, assignment of chroma and a tonal centroid to each window. After that, the tonal distance between consecutive frames is calculated based on a hypertoroid model of tonal space proposed by Chew (2000).

**Figure 2** gives a visual representation of each stimulus' spectrogram, an annotation of lyrics and chords or text and the time courses of the five extracted music features for a 12 s-segment.

### 2.5. Analysis

**Figure 1:**
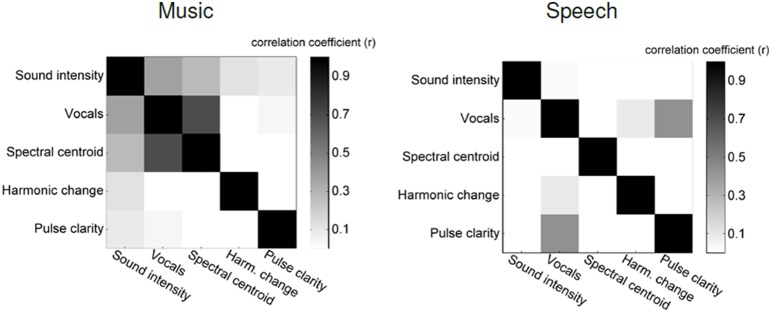
**Correlation between five stimulus features: left: music stimulus, right: speech stimulus**.

The five features that we used to describe the music stimulus are not independent of each other, but are correlated with each other to variable degrees (see Figure 1). Only by accounting for this correlation, one can attribute a particular ECoG signal to one particular music feature (Kendall et al., 1973). This *post-hoc* approach is a way to exert statistical control over variables in a setting where experimental control on the different aspects that are to be investigated is ruled out by design. The partial correlation coefficient is given by Equation (1).

(1) $$r_{xy.z}=\frac{r_{xy}-r_{xz}r_{yz}}{\sqrt{{\left(1-r_{xz}\right)}^{2}{\left(1-r_{yz}\right)}^{2}}}$$

Within the framework of linear regression analysis, the partial correlation coefficient can be derived as the correlation of the residuals that are produced if the interfering variable *z* (that is to be eliminated) is used as a regressor to predict each of the two variables of interest *x* and *y* (Abdi, 2007). The partial correlation coefficient is related to multiple linear regression analysis (MLR), which was applied in Schaefer et al. (2009) in a similar setting to decompose EEG responses into evoked response components that relate to specific aspects of music stimuli. Furthermore, regression-based models have been applied in a natural speech context in Power et al. (2012) where the auditory evoked spread spectrum analysis (AESPA) method gives a precise account of the temporal dynamics of the transformation of the speech envelope into an EEG signal in single trials. In Ding and Simon (2012) this is extended to an approach that differentiates further between a range of modulation frequencies of the stimulus signal, and, subsequently, identifies the slow temporal modulations of speech in a broad spectral region (below 1 Hz) as features that are represented best in the brain response. Importantly, the partial correlation coefficient differs in one important aspect from the semi-partial correlation/regression coefficient of the multiple linear regression framework: The partial correlation coefficient eliminates the influence of the interfering factor from both variables of interest, not only from one (in the framework of MLR: from the regressor). As a consequence, using the partial correlation coefficient, shared variance that does not cover a large proportion of the total variance, but may still reflect specific relations, is also detected. In a different context, partial correlation has been applied previously in connectivity analysis of EEG recordings: In Marrelec et al. (2006) it was used as a simple but effective method to identify connections between brain areas while accounting for the effects of volume conduction between electrodes. In contrast, here we examine how much each of the five features of music contributes to the sensor-level ECoG recordings in a manner that is independent from the remaining four features.

It is important to recognize that both ECoG features and the extracted music features have an autocorrelation, i.e., subsequent samples are not independent of each other. This fact violates the assumptions that underlie the standard tests for significance of correlation. To account for this issue, we assessed the significance of the partial correlation coefficients by applying randomized permutation tests with surrogate data as proposed in Theiler et al. (1992). For each music feature, we generated a surrogate target function by transforming the time domain signal into the frequency domain, randomly permuting its phase spectrum, and reconstructing the time domain signal using the original spectral amplitudes and the permuted phases. After that, we calculated the correlation coefficient between the ECoG feature and this surrogate target function. We repeated this process 1000 times, which resulted in a distribution of correlation coefficients for the surrogate data. We then asked how likely the observed correlation coefficient was to be produced by this surrogate distribution of correlation coefficients.

The resulting *p*-values were corrected for multiple comparisons within all electrodes [false discovery rate (FDR), *q* < 0.05]. We then plotted the negative logarithm of the corrected *p*-values for each electrode on each subject's brain model as an indicator of how much brain activity at a particular site was related to a specific acoustic feature. Since we did not observe negative correlation coefficients, there was no need to distinguish between negative and positive correlation.

Naturally, one would expect that a cortical brain response that tracks features of an auditory stimulus will not respond instantaneously, but delayed. Accordingly, we examined the channel-wise partial correlation coefficients with time lags up to 300 ms. However, this resulted in cross-correlation sequences that varied only on a very small scale over time and were not conclusive with respect to an optimal time lag, suggesting that a time lag between stimulus and brain response may be evened out by our sampling rate of 20 Hz. For instance, selecting a biologically plausible time lag of 100 ms, based on Kubanek et al. (2013) where the optimal (averaged) time lag for tracking the speech envelope ranged between 86.7 and 89.9 ms, had only an marginal effect on the significance of correlation coefficients, although the magnitude of correlation coefficients varied slightly (but not systematically). An overview of the group-level results for different time lags is depicted in Figure S3. On these grounds it would have been arbitrary to define a fixed time lag for the analysis and, moreover, a chosen time lag would not have been informative. Therefore, we decided to calculate instantaneous correlation coefficients in the present analysis, using this is a neutral or 'null' hypothesis given that no significant estimate of a biologically plausible time lag was obtainable for this data set. For a detailed analysis of latencies, in particular with respect to differences in the processing of different aspects of music, as suggested in Schaefer et al. (2011b), our approach is not appropriate since the dependencies between the five features play a role in calculating the partial correlation coefficients for one music feature and the ECoG signal. This could be a topic for a future investigation, for instance applying methods proposed in Bießmann et al. (2010) or Power et al. (2012).

Since these measures of significance cannot be directly averaged across subjects, to examine the topographical distribution of significant correlations at the group-level, we visualized the results as following: for each subject, we determined electrodes with significant correlation and projected their positions onto the MNI brain. To detect activated electrodes in similar regions, each of these electrodes was represented by a round patch of activation with radius 10 mm centered around its position. These representations were added up for the 10 subjects, resulting in a map showing the topographical overlap of the presence of significant correlation within the group of subjects. Values range from zero (no significant correlation in all ten subjects) to ten (significant correlation in all ten subjects). The degree of overlap is determined by the radius around an electrode (10 mm). Since grid placement was determined by clinical requirements and, consequently, varied between patients, we needed to account for the fact that the maximal number of subjects who can contribute to the group-level overlap of activation also varies between brain regions. Therefore, we determined the group-level overlap of grid coverage on the MNI brain, referred to as grid coverage index in the following, for all electrodes. Using the grid coverage index, a normalized group-level overlap in a specific cortical area can be obtained by dividing the (unnormalized) group-level overlap by the grid coverage index for each vertex. However, even the normalized group-level overlap values cannot be used for inferring group-level statistics, for instance to assess differences between brain areas. Nonetheless, this does not affect the primary goal of the present analysis, which is to explore potential differences in one location between features and also between the conditions music and pure speech. For distinct foci of high degree of group-level overlap, we determined representative coordinates on the MNI brain manually, and derived the corresponding Brodmann areas using the Talairach Atlas daemon^1^. Owing to the variance introduced by the projection of each subject's individual brain onto the MNI brain and to the blurring effect that the above mentioned procedure of determining group-level overlap may cause, this procedure yields only an approximate localization of cortical activation. Notwithstanding, on the scale of the Brodman area, this level of exactness appear appropriate for comparing the present results with the literature.

## 3. Results

Figure 1 shows a confusion matrix. For each element in this matrix, the brightness gives the correlation between two particular music features. In the music stimulus vocals on/off is strongly correlated with spectral centroid (*r* = 0.69) and intensity (*r* = 0.37), which confirms the necessity for calculating partial correlations.

**Figure 2:**
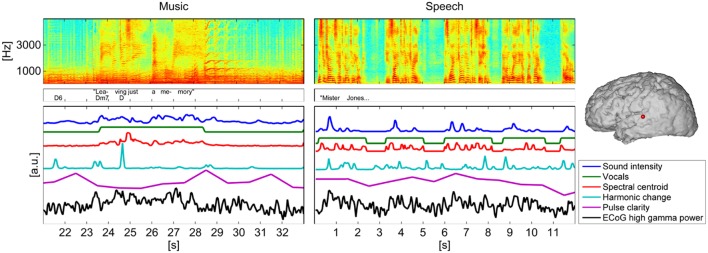
**Spectrogram of a segment (12 s) of the music/speech recording, lyrics/text, and chord annotations and time courses of the five analyzed features**. For comparison with the time course of the music features the time course of ECoG high gamma power, measured at one representative electrode of subject S5 was added below. The location of the electrode is indicated on the brain model on the right panel.

Figure 2 gives a visual representation of each stimulus' spectrogram, an annotation of lyrics and chords or text and the time courses of the five extracted music features for a 12 s-segment as well as the time course of ECoG high gamma power, measured at one representative electrode in one subject.

**Figure 3:**
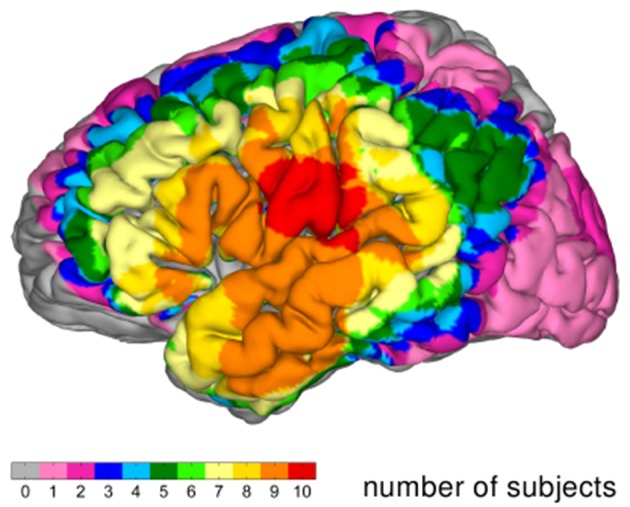
**Grid coverage index: Overlap of grid coverage on MNI brain**.

Figure 3 documents the overlap of grid coverage (grid coverage index) within the group of patients. The regions covered in all of the 10 subjects comprise the posterior part of the superior temporal gyrus and the ventral parts of the precentral and postcentral gyri.

**Figure 4:**
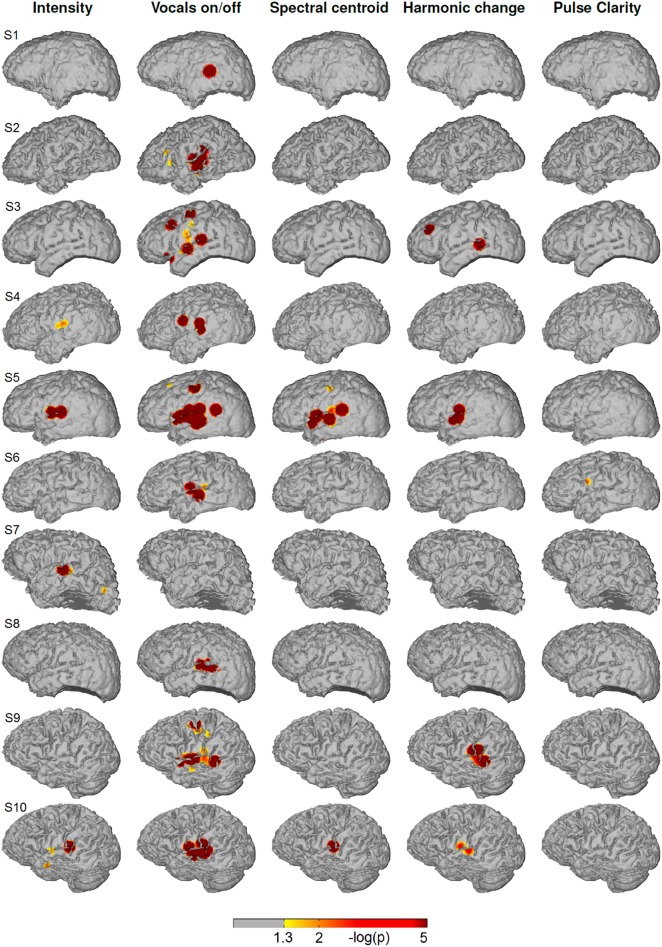
**Single subjects (individual brain models), music condition: Cortical distribution of significant correlation with each of the five acoustic features after removing the influence of the remaining four features by calculating partial correlation coefficients**. A value of 2 corresponds to a *p*-value of 0.01. Correlation coefficients determined as significant by permutation tests ranged between *r* = 0.07 and *r* = 0.26.

Figure 4 shows the significance values of partial correlation of ECoG high-gamma features with each of the five music features for each individual patient. Significant high-gamma correlations with vocals on/off are present in 9/10 of the subjects, and exceed in spatial extent those of all other acoustic features. In all of these nine patients, significant positive correlations are present in auditory areas around the Sylvian fissure, either confined to a region on the posterior superior temporal gyrus (pSTG) (S1, S2, S4, S6, and S8), or extending also to the anterior part of the STG and dorsally from the Sylvian fissure (S3, S5, S9, and S10). In addition, significant correlation in an isolated area at the dorsal precentral cortex is present in three subjects (S3, S5, and S9). Compared to the effect related to vocals on/off, correlation with sound intensity (after calculating the partial correlation and thereby rendering it now independent from fluctuations in the other four acoustic parameters, including vocals on/off) is low, reaching significance only in subject S4, S5, S7, and S10) and is detected only in a smaller region on the posterior STG. Correlation with spectral centroid is significant only in subject S5 and S10 and distributed similarly to the feature vocals on/off, but spatially less extended. For harmonic change, significant correlation is present in four subjects (subject S3, S5, S9, and S10) on the posterior STG and in subject S3 in frontal areas. The correlation with pulse clarity reaches significance in only one subjects (S6) in a small region on the precentral cortex.

**Figure 5:**
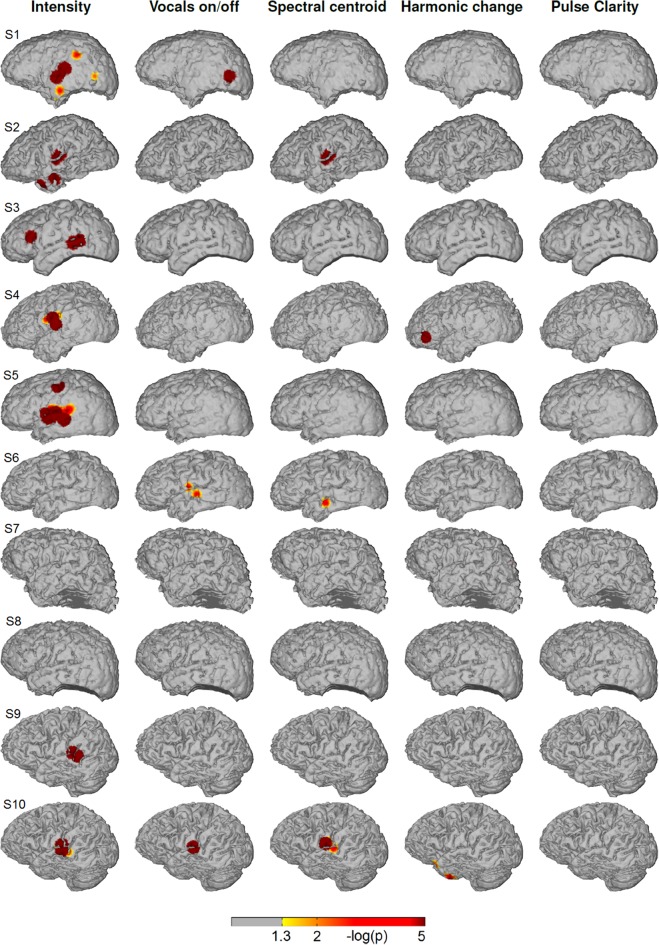
**Single subjects (individual brain models), speech condition: Cortical distribution of significant correlation with each of the five acoustic features after removing the influence of the remaining four features by calculating partial correlation coefficients**. A value of 2 corresponds to a *p*-value of 0.01. Correlation coefficients determined as significant by permutation tests ranged between *r* = 0.06 and *r* = 0.16.

Figure 5 depicts the cortical distribution of significant partial correlation of ECoG high-gamma features with each of the five acoustic features for the natural speech stimuli at the level of each individual patient. Differing from the music condition, the feature that is reflected most consistently within the group is sound intensity with significant correlation in 6/10 subjects (S1, S2, S3, S4, S5, S9, and S10). In all of them, the focus of correlation is located on the pSTG. Beyond that, significant correlation is present on the inferior/medial temporal gyrus (S1, S2), on the inferior frontal gyrus (S3) and on the precentral cortex (S5). For the feature spectral centroid, significant correlations are present only in three subjects on the superior and medial temporal gyrus. Of these, subject S10 is the only subject with significant correlation for spectral centroid in, both the music and the speech condition. For harmonic change, significant correlation is present only in subject S4 on the inferior frontal gyrus (IFG). For pulse clarity, no significant correlation with ECoG high gamma features is present.

**Figure 6:**
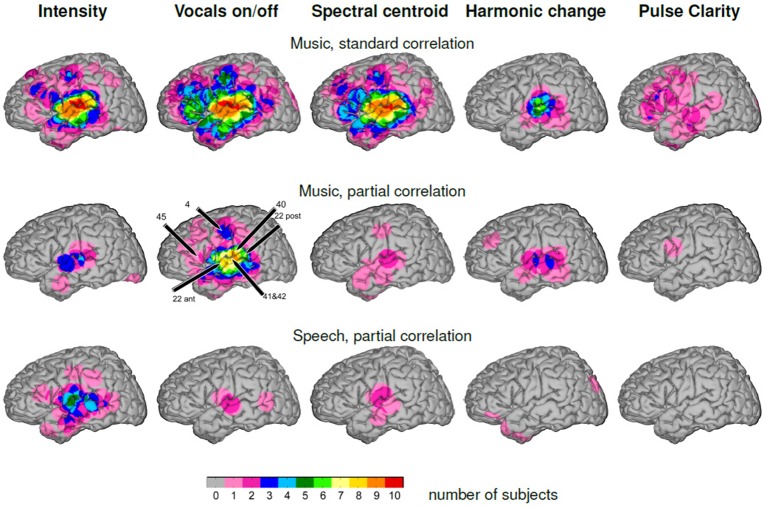
**Number of participants with effects visualized on the MNI brain**. The color code indicates the degree of group-level overlap. **Top:** Music, “standard” correlation. **Middle:** Music, partial correlation. **Bottom:** Speech, partial correlation.

The top row of Figure 6 shows the group-level overlap of significant “standard” correlation (Pearson's correlation coefficient without partialing out the other features) of high-gamma ECoG features with each of the five music features, i.e., including influences of the other features, on the MNI brain. Common to all patterns except pulse clarity is a focus of significant correlation in peri-Sylvian areas that is present in all ten subjects for the features sound intensity, vocals on/off, spectral centroid, in six subjects for harmonic change. The pattern for pulse clarity is most extended, and shows a large spatial variability of activation that do not overlap in more than three patients. In general, at a descriptive level, the similarity between cortical overlap patterns mirrors the correlation matrix of the music features in that they mainly document the interdependence of musical features rather than allowing to differentiate between processing of specific dimensions of music.

The middle row of Figure 6 gives the group-level overlap of significant correlation of high-gamma ECoG features with each of the five music features after the influence of the remaining four other features has been removed by calculating partial correlations (see Materials and Methods). The highest degree of overlap is present in the feature vocals on/off with significant correlation of high-gamma power with vocals on/off in more than seven subjects around the Sylvian fissure, covering the posterior and middle part of the superior temporal gyrus and of the middle temporal gyrus. The point of most consistently detected activations in the present group of subjects is the posterior part of the superior temporal gyrus (9/10 subjects). Furthermore, overlap of significant correlation is present in the precentral gyrus in three subjects. For all other features, the group-level overlap is considerably less: for sound intensity, there is a common focus of activation in the anterior peri-Sylvian area in three patients. Locations of significant correlation for harmonic change vary along the STG, amounting to a number of three overlapping subjects at the maximum. Significant correlation with spectral centroid is distributed around the Sylvian fissure, however with minimal inter-individual overlap.

The bottom row of Figure 6 shows the group-level overlap of significant correlation for complementary analysis of speech-only stimuli. The overlap of significant correlation with sound intensity is distributed around the Sylvian fissure with highest values on the middle part of the STG, corresponding to the respective location in the music condition, but with five contributing subjects, compared to three subjects in the music condition. However, for all other features the degree of overlap does not exceed two subjects in any location.

Figure S2 shows the group-level overlap depicted in Figure 6, normalized with respect to the grid coverage index depicted in Figure 3. We included only cortical sites with a minimum grid coverage of 2 subjects. This representation demonstrates that the characteristic patterns of the group-level overlap representation (Figure 5) do not merely reflect the distribution of the grid coverage index, but that the distribution of significant correlation has features that are consistently present in a large proportion of the subjects in which grid coverage is given.

Figure S3 shows the group-level overlap of significant correlation for delays of 0, 50, 100, 150, 200, 250, and 300 ms of the time course of the ECoG high gamma power and the music features.

## 4. Discussion

The present study explored the processing of complex natural music by examining relations between ECoG high-gamma band power and five features of music and thereby extends findings by Potes et al. (2012). To varying degree, these features co-fluctuate in original (unmodified) music and were found to produce similar cortical distributions of significant correlation in the high-gamma band. To address this issue, we calculated partial correlation coefficients to assess the unique impact of each of the five features (for comparison of standard and partial correlation see Figure 6, top and middle). Significant correlation of the high-gamma band ECoG features with the feature vocals on/off (indicating the change between purely instrumental passages and those with lyrics content) exceeded by far any of the other features in spatial extent and was consistently present within the group of subjects. Furthermore, distinct cortical patterns of significant correlation with the features sound intensity, spectral centroid and harmonic change were present in single subjects.

### 4.1. Reflection of aspects of music in ECoG high gamma power

These results demonstrate that in this example of a rock song, the change between purely instrumental passages and those with vocal lyrics content is the compositional feature that exerts the most marked effect on the electrocorticographic brain response in the high-gamma frequency band. In nine of ten patients, significant correlation of the high-gamma ECoG features with vocals on/off was present on the superior temporal gyrus. This led to a core region of high group-level overlap of significant correlation along the middle and posterior superior temporal gyrus (including Brodman areas 22, 41, and 42, see Figure 6, second column), particularly, extending posteriorly toward Wernicke's area (posterior BA 22 and BA 40) and also dorsally from the Sylvian fissure. In three subjects, significant correlation was also present on the dorsal precentral cortex (BA 4) and in two subjects on the inferior frontal gyrus near Broca's area (BA 45). Thus, the very onset of speech-related vocal content within a stream of music has a major impact on the brain response, as it affects stimulus-related neural activity distributed across several distinct brain regions. Considering that the partial correlation approach has removed the influence of the co-fluctuating four other factors that relate to sound intensity, to timbral (spectral centroid) or rhythmic characteristics (pulse clarity), and to harmonic structure, the remaining significant correlation could be related to speech-related aspects not addressed specifically in the analysis, such as linguistic-semantic aspect or the presence of the human voice that has been found to effect ECoG gamma activity even for (unintelligible) reversed speech in Brown et al. (2014). A more specific interpretation of this effect cannot be derived in the present context of a naturalistic complex music stimulus. The topography of the speech-related neural activity during listening to music is in line with Merrill et al. (2012), where the left superior temporal gyrus was found to code for the difference between (normal) speech and speech without words (hummed speech prosody) as well as for the difference between song with lyrics and song without lyrics (hummed). Furthermore, a differential BOLD response of the STG (bilaterally) for music with lyrics vs. instrumental music was observed in Brattico et al. (2011). The left mid-superior temporal sulcus was found to reflect (variable degrees of) integrated processing of lyrics and tunes (Sammler et al., 2010).

An activation of the dorsal precentral cortex in auditory perception of singing/speaking as well as in covert production has been observed before (Callan et al., 2006; Sammler et al., 2010) and was associated either with internal singing, or, more generally, with an activation of (pre)motor codes upon perception of song or speech. In particular, since the presentation of a full-length well-known rock song resembles a natural listening experience at least with respect to the stimulus material, one may speculate that patients might have silently sung along.

Beyond the impact of vocals on/off, a specific reflection of the features spectral centroid and harmonic change is present on the STG. For the spectral centroid, foci of significant correlation are present in 2/10 subjects (Figure 4). In both of them, they are located on the posterior part of the STG, which includes a part of Heschl's gyrus (BA 41 and 42). This particular area has been related to auditory processing in general, but specifically also to frequency (Liebenthal et al., 2003), pitch (Patterson et al., 2002), and harmonic tones (Zatorre and Krumhansl, 2002). The reflection of the spectral centroid on the STG is in line with Alluri et al. (2012) where the fluctuation of brightness (a component of timbre) in a natural music stimulus correlated with the BOLD response in the STG/MTG. For harmonic change, the focus of significant correlation is distributed similarly to spectral centroid extending also to Wernicke's area (posterior part of BA 22).

For sound intensity, significant correlation is present in 4/10 subjects. The maximal group-level overlap of significant correlation is located on the anterior STG.

Since pulse clarity was found to be reflected in the listener's BOLD response very clearly in the bi-lateral STG, insula and supplementary motor areas in Alluri et al. (2012), detecting a related reflection in the ECoG high gamma response would in principle have been possible with the present setup. Even though pulse clarity is (relatively within our feature set) uncorrelated with the other four features, significant correlation was present in only one subject and was confined to a small area. This suggests that changes in pulse clarity did not have a specific reflection in the ECoG high gamma response. A speculative explanation for the absence of rhythm-related effects in the brain response might include that acoustic features that have been found to be typical for “high-groove” music, such as clear pulses, high energy in low frequency bands, and high beat salience (Madison et al., 2011) may not be very salient in Pink Floyd's The Wall, part 1 (not to be confused with Pink Floyd's The Wall, part 2 that has strong drums).

### 4.2. Complementary analysis of speech stimuli

Considering that speech and music can both be characterized by similar concepts relating to temporal structure (onset structure and syllable structure), pitch-related structure (melody and prosody) and timbral aspects, it is an interesting question whether an identical description of a speech stimulus leads to similar reflection of extracted acoustic features in the high-gamma band. Therefore, we applied the same analysis to ECoG recordings of stimulation with natural speech [four narratives which are part of the Boston Aphasia Battery (Goodglass et al., 1983), for details see Kubanek et al., 2013 where this data set was analyzed previously].

In the speech condition, the feature vocals on/off was reflected much less than in the music condition, in two subjects on the superior temporal gyrus (STG) and in one subject in the posterior part of the middle lateral temporal lobe. Contrastingly, reflections of sound intensity were present in seven subjects with a common focus on the middle part of the STG and individually different distributed effects on the posterior part of the STG, on the MTG, on the dorsal precentral cortex, and on the inferior frontal gyrus.

One the one hand, the present distinct cortical reflection of temporal information in speech agrees with the essential role of the sound envelope in speech understanding that has been established by clinical results (Rosen, 1992; Drullman et al., 1994; Zeng et al., 1999; Lorenzi et al., 2006). On the other hand, the considerably weaker reflection in music suggests that, if speech-related content is embedded as song in music, the impact of this feature may be overruled by the change from instrumental to vocal/lyrics sound. Together with the fact that the contrast between silence and speech in the speech condition effected less change-related high gamma activity, this may indicate that the pSTG (where vocals on/off in the music condition was reflected most consistently), is responsive to vocals/lyrics in the context of music. Thus, it may be associated with identifying vocal speech-related content within the context of other complex sounds, reflecting a specific aspect of auditory scene analysis.

In the speech condition, for spectral centroid the group-level overlap of significant correlation is located on the pSTG, similarly to that in the music condition, but (apart from subject S10) different subjects contribute. This suggests that fluctuation of the spectral centroid is reflected on the posterior STG consistently in both conditions in single subjects, but, at the same time, that it is individually different for which stimulus and in whom these reflections reach significance.

In the speech condition, a reflection of the feature harmonic change is present in ECoG high-gamma power in one subject only while in the music condition 4/10 subjects showed a focus of significant correlation on the pSTG. The presence of an effect in the music condition demonstrates that the model-based extraction of harmonic change in music indeed has a physiological reflection. Notably, the harmonic change algorithm is tailored to measure the distance of chords within a tonal space, a metric that is not necessarily applicable to speech sounds. However, since speech also contains harmonic content, we hypothesized that, e.g., changes in the voice fundamental (F0) are extracted by the algorithm to some extent and may lead to a similar representation in the ECoG high gamma-power. Accordingly, the application of the harmonic change algorithm to the speech stimulus resulted in a time course showing a variance comparable to that in the music condition. The absence of such a representation in ECoG high gamma response (with exception of one subject) suggests that the music-specific extraction of harmonic features from the speech signal does not convey information triggering co-varying cortical processing.

To summarize, in this differential analysis of five features of a natural music stimulus, we found the on/offset of vocal lyrics to be the dominant driver of ECoG high-gamma power on the STG (mostly in the posterior part) and in peri-Sylvian areas consistently within the group of subjects. In parallel, in single subjects, sound intensity, harmonic change, and spectral centroid produced specific high gamma reflections in the same brain area. In the speech condition, topographically similar effects for sound intensity were present most consistently, and in single subjects for vocals on/off, harmonic change and spectral centroid. In general, these findings are in line with the assumed involvement of the pSTG in the intermediate stage of auditory processing (Pasley et al., 2012), more specifically in the selective extraction of spectro-temporal features relevant for auditory object recognition. Thus, the observed different activations between music and speech may demonstrate differences in the relative importance of the features in both stimuli. Tentatively, one may explain the inter-individual differences with respect to the presence of effects for harmonic change, spectral centroid and sound intensity with different contributions of the five features to an individual's listening experience.

The present results differentiate further the pioneering work of Potes et al. (2012) where ECoG high-gamma features were found to trace sound intensity in two distinct regions, in the posterior STG and in the inferior frontal gyrus. The present follow-up analysis helps to attribute this effect mainly to the presence of vocal speech in the stimulus while the effect of sound intensity proper is found much weaker and confined to a smaller region on the STG. In addition, with spectral centroid and harmonic change, we identified two further aspects specific for music that have an impact on the high-gamma ECoG response in some subjects. Notwithstanding, in these single subjects, these effects are highly significant and derived from one presentation of the stimulus.

The present results complement those of Kubanek et al. (2013) where high-gamma ECoG activity was reported to track the temporal envelope of natural speech stimuli in non-primary areas of the auditory cortex: in the superior temporal gyrus (STG) and on the inferior frontal gyrus (IFG) near Broca's area. On the other hand, the temporal envelope of music including song (condition “lyrics,” represented by the concatenated periods of singing) and purely instrumental music (condition “melody,” represented by the purely instrumental periods) was tracked considerably weaker by high-gamma ECoG activity and only in the auditory belt area. This suggested a specificity of STG and IFG for speech-related processing and confirmed the importance of temporal information in speech. The present results help to further elucidate the previous results insofar that they demonstrate that not only the sound envelope is encoded weaker in the music condition, but that the alternating presence/absence of vocals is represented predominantly.

Since research on neural processing of natural music is heterogeneous with respect to data-recording techniques, stimulus material, music features, and methods of data analysis, it is difficult to directly compare results of different studies. With respect to physiology, the present data reveal that in addition to the alpha and theta frequency bands, which have been found to reflect dynamic rhythmic features of music (Cong et al., 2012), the high-gamma band carries information about the music stimulus.

Our analysis was focused on the high gamma frequency range, based on initial results that were not informative in lower frequency bands. Negative correlation between ECoG power in lower frequency bands (8–12 and 18–24 Hz) and sound intensity was reported in Potes et al. (2012). Recently, Potes et al. (2014) showed in a detailed analysis that envelope-related high gamma activity (70–110 Hz) in areas close to primary auditory cortex, in peri-Sylvian and superior pre-motor areas precedes and predicts envelope-related alpha band activity (8–12 Hz) near primary auditory areas that have been found to be the target of afferent auditory projections from the thalamus. In light of the hypothesis of Kumar et al. (2007) and Zatorre et al. (2007) the alpha band activity has been associated with relay mechanisms that govern the transfer of auditory information from the thalamus to core auditory areas. The high gamma activity has been related to cortical extraction of complex auditory features in non-primary auditory areas. According to this model, the high gamma frequency range would be suitable starting point for a differential analysis of the processing of higher-level auditory features. However, extending the present analysis to lower frequency bands in the future could provide additional information. In a natural speech context, in particular phase information in low-frequency bands of the brain signal has been found to be informative in Zion Golumbic et al. (2013) and Ding and Simon (2012).

### 4.3. Methodology

With respect to methodology, we approach a typical problem that arises when assessing the relationship between brain recordings and natural auditory stimuli. We address the problem of interdependent features of natural complex stimuli that complicate the correlation-based analysis of the relation between brain signals and stimulus features. Recently, in Alluri et al. (2013), this challenge has been faced by applying principal component regression modeling where the interrelated multi-dimensional description of a music signal is transformed into a lower-dimensional space of uncorrelated components that are subsequently perceptually evaluated. Here, operating on the original features, we demonstrate that a partial correlation approach as an extension of multiple linear regression analysis (Schaefer et al., 2009) can be used to differentiate between the processing of aspects of natural music.

Typically, in a naturalistic setting, multi-channel measurements are related (e.g., by correlation measures) to a multi-dimensional description of music, a situation which is prone to produce false positive effects such as spurious correlations. One way of constraining the solution is to assume inter-individual consistent spatial distribution of neural activity, e.g., by averaging the EEG time course across subjects (Schaefer et al., 2009) or by selecting components that are common to the majority of subjects (Alluri et al., 2012; Cong et al., 2012). The present results are an important complement to previous studies, as they were obtained at the single-subject level and for one single stimulus presentation owing to the ECoG's characteristics of offering both high temporal and spatial resolution. This sensitivity helped to reveal a considerable variability between subjects with respect to reflected features of music, an insight that suggests that assuming within-group consistency might neglect some of these individual effects.

### 4.4. Current experimental limitations

Obviously, there are limitations of what can be achieved with this approach. Typical for ECoG recordings, the data were recorded from epilepsy patients whose physical and cognitive conditions were impaired to different degrees and whose brains may not be comparable to that of the healthy population in function and neuroanatomy. Furthermore, grid coverage was determined by clinical reasons and thus varied between subjects. Important issues, such as hemispheric specialization for speech and music, cannot be addressed with the present data set of left-hemispheric recordings. Another important issue is, that information about the patients' music preference, cultural background and musical training that could give valuable clues for interpreting inter-personal differences is not available in this follow-up analysis.

## 5. Conclusion

However, our analysis is an example of what can be achieved within these limits and contributes to the growing body of methodological approaches for research on the processing of natural music. Partial correlation, proposed here as one solution for inter-dependence of stimulus features, has detected specific reflections of music features in the ECoG high-gamma response. However, it has to be kept in mind that this method gives a differential picture of each music feature's impact on the brain response showing cortical reflections that are unique to this feature beyond all others in the feature set. Thus, for a given feature, the portion of independent variance from the other features is crucial for the detectability of its reflection in the brain response. It should be kept in mind that the present approach provides a differential view on brain responses to aspects of a natural music stimulus, not a comprehensive decomposition of the brain signal.

Naturally, when comparing two different stimuli, such as in our case in the speech and music condition, the individual interdependence of stimulus features is not the same, nor can the stimulus features themselves be balanced between both stimuli. Our results, therefore, have to be regarded as highly specific cortical imprints of two different, naturally unbalanced examples of natural auditory stimulation from two sound categories, not as general findings on the processing of music or speech. Nonetheless, the present differentiated picture of brain responses at the level of single subjects and a single presentation is a valuable complement of the recent series of investigations in natural music processing research.

### Conflict of interest statement

The authors declare that the research was conducted in the absence of any commercial or financial relationships that could be construed as a potential conflict of interest.

## Footnotes

^1^http://www.talairach.org/daemon.html.

## References

[B1] Abdi H. (2007). Part (semi partial) and partial regression coefficients, in Encyclopedia of Measurement and Statistics, ed Salkind N. J. (SAGE Publications), 736–740

[B2] Abrams D. A. Bhatara A. Ryali S. Balaban E. Levitin D. J. Menon V. (2011). Decoding temporal structure in music and speech relies on shared brain resources but elicits different fine-scale spatial patterns. Cereb. Cortex 21, 1507–1518 10.1093/cercor/bhq198 21071617 PMC3116734

[B3] Abrams D. A. Ryali S. Chen T. Chordia P. Khouzam A. Levitin D. J. (2013). Inter-subject synchronization of brain responses during natural music listening. Eur. J. Neurosci. 37, 1458–1469 10.1111/ejn.12173 23578016 PMC4487043

[B4] Alluri V. Toiviainen P. Jääskeläinen I. P. Glerean E. Sams M. Brattico E. (2012). Large-scale brain networks emerge from dynamic processing of musical timbre, key and rhythm. Neuroimage 59, 3677–3689 10.1016/j.neuroimage.2011.11.019 22116038

[B5] Alluri V. Toiviainen P. Lund T. E. Wallentin M. Vuust P. Nandi A. K. (2013). From vivaldi to beatles and back: predicting lateralized brain responses to music. Neuroimage 83, 627–636 10.1016/j.neuroimage.2013.06.064 23810975

[B6] Bießmann F. Meinecke F. C. Gretton A. Rauch A. Rainer G. Logothetis N. K. (2010). Temporal kernel cca and its application in multimodal neuronal data analysis. Mach. Learn. 79, 5–27 10.1007/s10994-009-5153-3

[B7] Brattico E. Alluri V. Bogert B. Jacobsen T. Vartiainen N. Nieminen S. (2011). A functional MRI study of happy and sad emotions in music with and without lyrics. Front. Psychol. 2:308 10.3389/fpsyg.2011.00308 22144968 PMC3227856

[B8] Brattico E. Tervaniemi M. Naatanen R. Peretz I. (2006). Musical scale properties are automatically processed in the human auditory cortex. Brain Res. 1117, 162–174 10.1016/j.brainres.2006.08.023 16963000

[B9] Brown E. C. Muzik O. Rothermel R. Juhász C. Shah A. K. Fuerst D. (2014). Evaluating signal-correlated noise as a control task with language-related gamma activity on electrocorticography. Clin. Neurophysiol. 125, 1312–1323 10.1016/j.clinph.2013.11.026 24412331 PMC4035421

[B10] Burger B. Thompson M. R. Luck G. Saarikallio S. Toiviainen P. (2013). Influences of rhythm-and timbre-related musical features on characteristics of music-induced movement. Front. psychol. 4:183 10.3389/fpsyg.2013.00183 23641220 PMC3624091

[B11] Caclin A. Brattico E. Tervaniemi M. Näätänen R. Morlet D. Giard M.-H. (2006). Separate neural processing of timbre dimensions in auditory sensory memory. J. Cogn. Neurosci. 18, 1959–1972 10.1162/jocn.2006.18.12.1959 17129184

[B12] Caclin A. Giard M.-H. Smith B. K. McAdams S. (2007). Interactive processing of timbre dimensions: a garner interference study. Brain Res. 1138, 159–170 10.1016/j.brainres.2006.12.065 17261274

[B13] Callan D. E. Tsytsarev V. Hanakawa T. Callan A. M. Katsuhara M. Fukuyama H. (2006). Song and speech: brain regions involved with perception and covert production. Neuroimage 31, 1327–1342 10.1016/j.neuroimage.2006.01.036 16546406

[B14] Chapin H. Jantzen K. Kelso J. Steinberg F. Large E. (2010). Dynamic emotional and neural responses to music depend on performance expression and listener experience. PLoS ONE 5:e13812 10.1371/journal.pone.0013812 21179549 PMC3002933

[B15] Chew E. (2000). Towards a Mathematical Model of Tonality. Ph.D. thesis, Massachusetts Institute of Technology.

[B16] Cong F. Phan A. H. Zhao Q. Nandi A. K. Alluri V. Toiviainen P. (2012). Analysis of ongoing EEG elicited by natural music stimuli using nonnegative tensor factorization, in Signal Processing Conference (EUSIPCO), 2012 (Bucharest), 494–498

[B17] Coutinho E. Cangelosi A. (2011). Musical emotions: predicting second-by-second subjective feelings of emotion from low-level psychoacoustic features and physiological measurements. Emotion 11, 921–937 10.1037/a0024700 21859207

[B18] Crone N. E. Sinai A. Korzeniewska A. (2006). High-frequency gamma oscillations and human brain mapping with electrocorticography. Prog. Brain Res. 159, 275–295 10.1016/S0079-6123(06)59019-3 17071238

[B19] Daikoku T. Ogura H. Watanabe M. (2012). The variation of hemodynamics relative to listening to consonance or dissonance during chord progression. Neurol. Res. 34, 557–563 10.1179/1743132812Y.0000000047 22642826

[B20] Deike S. Gaschler-Markefski B. Brechmann A. Scheich H. (2004). Auditory stream segregation relying on timbre involves left auditory cortex. Neuroreport 15, 1511–1514 10.1097/01.wnr.0000132919.12990.34 15194885

[B21] del Bimbo A. Chang S.-F. Smeulders A. Cannam C. Landone C. Sandler M. (2010). Sonic visualiser, in Proceedings of The International Conference on Multimedia - MM '10 (Firenze: ACM Press), 1467

[B22] Ding N. Simon J. Z. (2012). Neural coding of continuous speech in auditory cortex during monaural and dichotic listening. J. Neurophysiol. 107, 78–89 10.1152/jn.00297.2011 21975452 PMC3570829

[B23] Drullman R. Festen J. M. Plomp R. (1994). Effect of reducing slow temporal modulations on speech reception. J. Acoust. Soc. Am. 95, 2670 10.1121/1.409836 8207140

[B24] Edwards E. Soltani M. Deouell L. Y. Berger M. S. Knight R. T. (2005). High gamma activity in response to deviant auditory stimuli recorded directly from human cortex. J. Neurophysiol. 94, 4269–4280 10.1152/jn.00324.2005 16093343

[B25] Eerola T. Lartillot O. Toiviainen P. (2009). Prediction of multidimensional emotional ratings in music from audio using multivariate regression models, in Proceedings of ISMIR (Kobe), 621–626

[B26] Goodglass H. Edith K. Barbara B. (1983). BDAE: The Boston Diagnostic Aphasia Examination. Philadelphia, PA: Lea and Febiger

[B27] Goydke K. Altenmüller E. Möller J. Münte T. (2004). Changes in emotional tone and instrumental timbre are reflected by the mismatch negativity. Cogn. Brain Res. 21, 351–359 10.1016/j.cogbrainres.2004.06.009 15511651

[B28] Grahn J. A. Rowe J. B. (2009). Feeling the beat: premotor and striatal interactions in musicians and nonmusicians during beat perception. J. Neurosci. 29, 7540–7548 10.1523/JNEUROSCI.2018-08.2009 19515922 PMC2702750

[B29] Halpern A. Martin J. Reed T. (2008). An ERP study of major-minor classification in melodies. Music Percept. 25, 181–191 10.1525/mp.2008.25.3.181

[B30] Harte C. Gasser M. Mark S. (2006). Detecting harmonic change in musical audio, in AMCMM '06 Proceedings of The 1st ACM Workshop on Audio and Music Computing Multimedia (Santa Barbara, CA), 21–26

[B31] Hasson U. Malach R. Heeger D. J. (2010). Reliability of cortical activity during natural stimulation. Trends Cogn. Sci. 14, 40–48 10.1016/j.tics.2009.10.011 20004608 PMC2818432

[B32] Higuchi M. Fornari J. Del Ben C. Graeff F. Leite J. P. (2011). Reciprocal modulation of cognitive and emotional aspects in pianistic performances. PLoS ONE 6:e24437 10.1371/journal.pone.0024437 21931716 PMC3170321

[B33] Hyde K. L. Peretz I. Zatorre R. J. (2008). Evidence for the role of the right auditory cortex in fine pitch resolution. Neuropsychologia 46, 632–639 10.1016/j.neuropsychologia.2007.09.004 17959204

[B34] Janata P. (2009). The neural architecture of music-evoked autobiographical memories. Cereb. Cortex 19, 2579 10.1093/cercor/bhp008 19240137 PMC2758676

[B35] Janata P. Birk J. van Horn J. Leman M. Tillmann B. Bharucha J. (2002). The cortical topography of tonal structures underlying Western music. Science 298, 2167 10.1126/science.1076262 12481131

[B36] Jentschke S. Friederici A. D. Koelsch S. (2014). Neural correlates of music-syntactic processing in two-year old children. Dev. Cogn. Neurosci. 9, 200–208 10.1016/j.dcn.2014.04.005 24907450 PMC6989737

[B37] Jongsma M. L. Desain P. Honing H. (2004). Rhythmic context influences the auditory evoked potentials of musicians and nonmusicians. Biol. Psychol. 66, 129–152 10.1016/j.biopsycho.2003.10.002 15041136

[B38] Kendall M. G. Stuart A. Ord J. K. (1973). Inference and Relationship, Volume 2 of The Advanced Theory of Statistics, 3rd Edn London: Griffin

[B39] Kim C. H. Lee S. Kim J. S. Seol J. Yi S. W. Chung C. K. (2014). Melody effects on eranm elicited by harmonic irregularity in musical syntax. Brain Res. 1560, 36–45 10.1016/j.brainres.2014.02.045 24607297

[B40] Koelsch S. Gunter T. C. von Cramon D. Y. Zysset S. Lohmann G. Friederici A. D. (2002). Bach speaks: a cortical “language-network” serves the processing of music. Neuroimage 17, 956–966 10.1006/nimg.2002.1154 12377169

[B41] Kubanek J. Brunner P. Gunduz A. Poeppel D. Schalk G. Rodriguez-Fornells A. (2013). The tracking of speech envelope in the human cortex. PLoS ONE 8:e53398 10.1371/journal.pone.0053398 23408924 PMC3542338

[B42] Kumar S. Sedley W. Nourski K. V. Kawasaki H. Oya H. Patterson R. D. (2011). Predictive coding and pitch processing in the auditory cortex. J. Cogn. Neurosci. 23, 3084–3094 10.1162/jocn_a_00021 21452943 PMC3821983

[B43] Kumar S. Stephan K. E. Warren J. D. Friston K. J. Griffiths T. D. (2007). Hierarchical processing of auditory objects in humans. PLoS Comput. Biol. 3:e100 10.1371/journal.pcbi.0030100 17542641 PMC1885275

[B44] Lartillot O. Eerola T. Toiviainen P. Fornari J. (2008a). Multi-feature modeling of pulse clarity: design, validation and optimization, in Proceedings of ISMIR (Philadelphia, PA: Citeseer), 521–526

[B45] Lartillot O. Toiviainen P. Eerola T. (2008b). A matlab toolbox for music information retrieval, in Studies in Classification, Data Analysis, and Knowledge Organization, eds Preisach C. Burkhardt H. Schmidt-Thieme L. Decker R. (Berlin; Heidelberg: Springer Berlin Heidelberg), 261–268

[B46] Lehne M. Rohrmeier M. Koelsch S. (2014). Tension-related activity in the orbitofrontal cortex and amygdala: an fmri study with music. Soc. Cogn. Affect. Neurosci. 9, 1515–1523 10.1093/scan/nst141 23974947 PMC4187266

[B47] Leonard M. K. Chang E. F. (2014). Dynamic speech representations in the human temporal lobe. Trends Cogn. Sci. 18, 472–479 10.1016/j.tics.2014.05.001 24906217 PMC4149812

[B48] Liebenthal E. Ellingson M. L. Spanaki M. V. Prieto T. E. Ropella K. M. Binder J. R. (2003). Simultaneous ERP and fMRI of the auditory cortex in a passive oddball paradigm. Neuroimage 19, 1395–1404 10.1016/S1053-8119(03)00228-3 12948697

[B49] Lorenzi C. Gilbert G. Carn H. Garnier S. Moore B. C. (2006). Speech perception problems of the hearing impaired reflect inability to use temporal fine structure. Proc. Natl. Acad. Sci. U.S.A. 103, 18866–18869 10.1073/pnas.0607364103 17116863 PMC1693753

[B50] Madison G. Gouyon F. Ullén F. Hörnström K. (2011). Modeling the tendency for music to induce movement in humans: first correlations with low-level audio descriptors across music genres. J. Exp. Psychol. Hum. Percept. Perform. 37, 1578 10.1037/a0024323 21728462

[B51] Marrelec G. Krainik A. Duffau H. Pélégrini-Issac M. Lehéricy S. Doyon J. (2006). Partial correlation for functional brain interactivity investigation in functional MRI. Neuroimage 32, 228–237 10.1016/j.neuroimage.2005.12.057 16777436

[B52] Martin S. Brunner P. Holdgraf C. Heinze H.-J. Crone N. E. Rieger J. (2014). Decoding spectrotemporal features of overt and covert speech from the human cortex. Front. Neuroeng. 7:14 10.3389/fneng.2014.00014 24904404 PMC4034498

[B53] Merrill J. Sammler D. Bangert M. Goldhahn D. Lohmann G. Turner R. (2012). Perception of words and pitch patterns in song and speech. Front. Psychol. 3:76 10.3389/fpsyg.2012.00076 22457659 PMC3307374

[B54] Mikutta C. Altorfer A. Strik W. Koenig T. (2012). Emotions, arousal, and frontal alpha rhythm asymmetry during beethoven's 5th symphony. Brain Topogr. 25, 423–430 10.1007/s10548-012-0227-0 22534936

[B55] Mikutta C. A. Schwab S. Niederhauser S. Wuermle O. Strik W. Altorfer A. (2013). Music, perceived arousal, and intensity: psychophysiological reactions to chopin's tristesse. Psychophysiology 50, 909–919 10.1111/psyp.12071 23763714

[B56] Nan Y. Friederici A. D. (2013). Differential roles of right temporal cortex and broca's area in pitch processing: evidence from music and mandarin. Hum. Brain Mapp. 34, 2045–2054 10.1002/hbm.22046 22431306 PMC6870388

[B57] Pampalk E. Rauber A. Merkl D. (2002). Content-based organization and visualization of music archives, in Proceedings of The Tenth ACM International Conference on Multimedia (Juan les Pins), 570–579 10.1145/641007.641121

[B58] Pasley B. N. David S. V. Mesgarani N. Flinker A. Shamma S. A. Crone N. E. (2012). Reconstructing speech from human auditory cortex. PLoS Biol. 10:e1001251 10.1371/journal.pbio.1001251 22303281 PMC3269422

[B59] Patterson R. D. Uppenkamp S. Johnsrude I. S. Griffiths T. D. (2002). The processing of temporal pitch and melody information in auditory cortex. Neuron 36, 767–776 10.1016/S0896-6273(02)01060-7 12441063

[B60] Pei X. Leuthardt E. C. Gaona C. M. Brunner P. Wolpaw J. R. Schalk G. (2011). Spatiotemporal dynamics of electrocorticographic high gamma activity during overt and covert word repetition. Neuroimage 54, 2960–2972 10.1016/j.neuroimage.2010.10.029 21029784 PMC3020260

[B61] Perani D. Saccuman M. Scifo P. Spada D. Andreolli G. Rovelli R. (2010). Functional specializations for music processing in the human newborn brain. Proc. Natl. Acad. Sci. U.S.A. 107, 4758 10.1073/pnas.0909074107 20176953 PMC2842045

[B62] Plack C. J. Barker D. Hall D. A. (2014). Pitch coding and pitch processing in the human brain. Hear. Res. 307, 53–64 10.1016/j.heares.2013.07.020 23938209

[B63] Potes C. Brunner P. Gunduz A. Knight R. T. Schalk G. (2014). Spatial and temporal relationships of electrocorticographic alpha and gamma activity during auditory processing. Neuroimage 97, 188–195 10.1016/j.neuroimage.2014.04.045 24768933 PMC4065821

[B64] Potes C. Gunduz A. Brunner P. Schalk G. (2012). Dynamics of electrocorticographic (ecog) activity in human temporal and frontal cortical areas during music listening. Neuroimage 61, 841–848 10.1016/j.neuroimage.2012.04.022 22537600 PMC3376242

[B65] Power A. J. Foxe J. J. Forde E.-J. Reilly R. B. Lalor E. C. (2012). At what time is the cocktail party? A late locus of selective attention to natural speech. Eur. J. Neurosci. 35, 1497–1503 10.1111/j.1460-9568.2012.08060.x 22462504

[B66] Regnault P. Bigand E. Besson M. (2001). Different brain mechanisms mediate sensitivity to sensory consonance and harmonic context: evidence from auditory event-related brain potentials. J. Cogn. Neurosci. 13, 241–255 10.1162/089892901564298 11244549

[B67] Rosen S. (1992). Temporal information in speech: acoustic, auditory and linguistic aspects. Philos. Trans. R. Soc. Lond. B Biol. Sci. 336, 367–373 10.1098/rstb.1992.0070 1354376

[B68] Sammler D. Baird A. Valabrègue R. Clément S. Dupont S. Belin P. (2010). The relationship of lyrics and tunes in the processing of unfamiliar songs: a functional magnetic resonance adaptation study. J. Neurosci. 30, 3572–3578 10.1523/JNEUROSCI.2751-09.2010 20219991 PMC6632242

[B69] Sammler D. Koelsch S. Ball T. Brandt A. Grigutsch M. Huppertz H.-J. (2013). Co-localizing linguistic and musical syntax with intracranial EEG. Neuroimage 64, 134–146 10.1016/j.neuroimage.2012.09.035 23000255

[B70] Sammler D. Koelsch S. Friederici A. D. (2011). Are left fronto-temporal brain areas a prerequisite for normal music-syntactic processing? Cortex 47, 659–673 10.1016/j.cortex.2010.04.007 20570253

[B71] Schaefer R. Vlek R. Desain P. (2011a). Decomposing rhythm processing: electroencephalography of perceived and self-imposed rhythmic patterns. Psychol. Res. 75, 95–106 10.1007/s00426-010-0293-4 20574661 PMC3036830

[B72] Schaefer R. S. Desain P. Suppes P. (2009). Structural decomposition of EEG signatures of melodic processing. Biol. Psychol. 82, 253–259 10.1016/j.biopsycho.2009.08.004 19698758

[B73] Schaefer R. S. Farquhar J. Blokland Y. Sadakata M. Desain P. (2011b). Name that tune: decoding music from the listening brain. Neuroimage 56, 843–849 10.1016/j.neuroimage.2010.05.084 20541612

[B74] Schalk G. McFarland D. J. Hinterberger T. Birbaumer N. Wolpaw J. R. (2004). BCI2000: a general-purpose brain-computer interface (bci) system. IEEE Trans. Biomed. Eng. 51, 1034–1043 10.1109/TBME.2004.827072 15188875

[B75] Schalk G. Mellinger J. (2010). Human-Computer Interaction: Practical Guide to Brain-Computer Interfacing with BCI2000: General-Purpose Software for Brain-Computer Interface Research, Data Acquisition, Stimulus Presentation, and Brain Monitoring. London, UK: Springer

[B76] Schubert E. Wolfe J. Tarnopolsky A. (2004). Spectral centroid and timbre in complex, multiple instrumental textures, in Proceedings of The International Conference on Music Perception and Cognition, North Western University, Illinois 2004 (Chicago, IL), 112–116

[B77] Snyder J. S. Large E. W. (2005). Gamma-band activity reflects the metric structure of rhythmic tone sequences. Cogn. Brain Res. 24, 117–126 10.1016/j.cogbrainres.2004.12.014 15922164

[B78] Theiler J. Eubank S. Longtin A. Galdrikian B. Doyne Farmer J. (1992). Testing for nonlinearity in time series: the method of surrogate data. Physica D 58, 77–94 10.1016/0167-2789(92)90102-S

[B79] Toiviainen P. Alluri V. Brattico E. Wallentin M. Vuust P. (2014). Capturing the musical brain with lasso: dynamic decoding of musical features from fmri data. Neuroimage 88, 170–180 10.1016/j.neuroimage.2013.11.017 24269803

[B80] Towle V. L. Yoon H.-A. Castelle M. Edgar J. C. Biassou N. M. Frim D. M. (2008). Ecog gamma activity during a language task: differentiating expressive and receptive speech areas. Brain 131, 2013–2027 10.1093/brain/awn147 18669510 PMC2724904

[B81] Trainor L. McDonald K. Alain C. (2002). Automatic and controlled processing of melodic contour and interval information measured by electrical brain activity. J. Cogn. Neurosci. 14, 430–442 10.1162/089892902317361949 11970802

[B82] Zatorre R. Chen J. Penhune V. (2007). When the brain plays music: auditory–motor interactions in music perception and production. Nat. Rev. Neurosci. 8, 547–558 10.1038/nrn2152 17585307

[B83] Zatorre R. Krumhansl C. (2002). Mental models and musical minds. Science 298, 2138 10.1126/science.1080006 12481121

[B84] Zeng F.-G. Oba S. Garde S. Sininger Y. Starr A. (1999). Temporal and speech processing deficits in auditory neuropathy. Neuroreport 10, 3429–3435 10.1097/00001756-199911080-00031 10599857

[B85] Zentner M. (2010). Homer's prophecy: an essay on music's primary emotions. Music Anal. 29, 102–125 10.1111/j.1468-2249.2011.00322.x

[B86] Zion Golumbic E. M. Ding N. Bickel S. Lakatos P. Schevon C. A. McKhann G. M. (2013). Mechanisms underlying selective neuronal tracking of attended speech at a “cocktail party”. Neuron 77, 980–991 10.1016/j.neuron.2012.12.037 23473326 PMC3891478

