# Supplementary material for: ECoG high gamma activity reveals distinct cortical representations of lyrics passages, harmonic and timbre-related changes in a rock song
Source: Front Hum Neurosci. 2014 Oct 13;8:798. doi: 10.3389/fnhum.2014.00798 (PMC4195312; doi:10.3389/fnhum.2014.00798)
Supplement: Supplementary file 1 [file Presentation1.PDF]

SUPPLEMENTAL DATA

Table1. Clinical profiles of the subjects that participated in the study. All subjects had normal cognitive capacity and were functionally independent.

Subject	Age	Sex	Handedness	Seizure Focus	Number of electrodes
1	29	F	R	Left temporal	86
2	30	M	R	Left temporal	82
3	26	F	R	Left temporal	103
4	45	M	R	Left temporal	56
5	29	F	R	Left temporal	108
6	45	F	L	Left temporal	57
7	60	M	R	Left temporal	53
8	17	F	L	Left temporal	93
9	28	M	R	Left temporal	110
10	25	F	R	Left temporal	92

Figure S1 shows the group-level overlap of significance of 'standard' correlation coefficients for the speech condition.

Figure S2 shows the group-level overlap depicted in Figure 6, normalized with respect to the grid coverage index depicted in Figure 3. This representation demonstrates that the characteristic patterns of the group-level overlap representation (Figure 6) do not merely reflect the distribution of the grid coverage index, but that the distribution of significant correlation has features that are consistently present in a large proportion of the subjects in which grid coverage is given.

Figure S3 shows the group-level overlap of significance of partial correlation, calculated for time delays of 0, 50, . . . , 300 ms of the time course of the ECoG high gamma power with respect to the music stimulus.
