# Supplementary figures and images for: ECoG high gamma activity reveals distinct cortical representations of lyrics passages, harmonic and timbre-related changes in a rock song
Source: Front Hum Neurosci. 2014 Oct 13;8:798. doi: 10.3389/fnhum.2014.00798 (PMC4195312; doi:10.3389/fnhum.2014.00798)

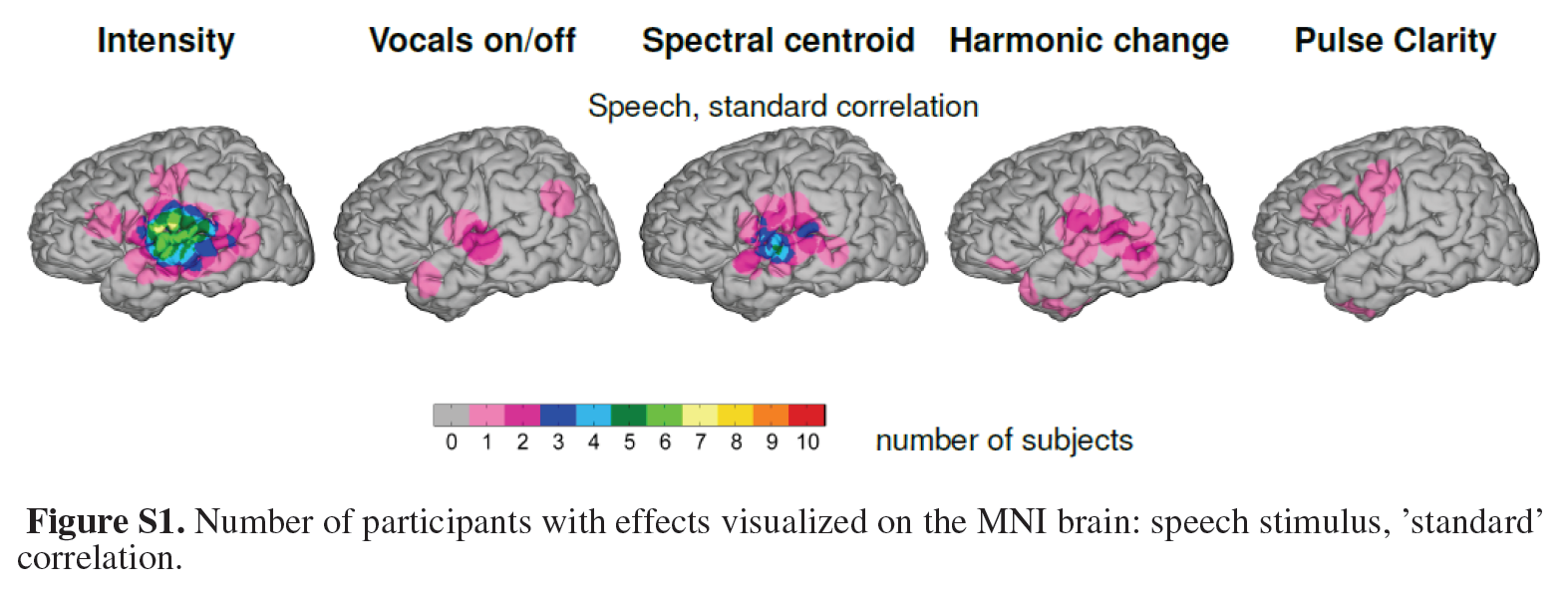

Supplement: Supplementary file 2 [file Image1.TIF]

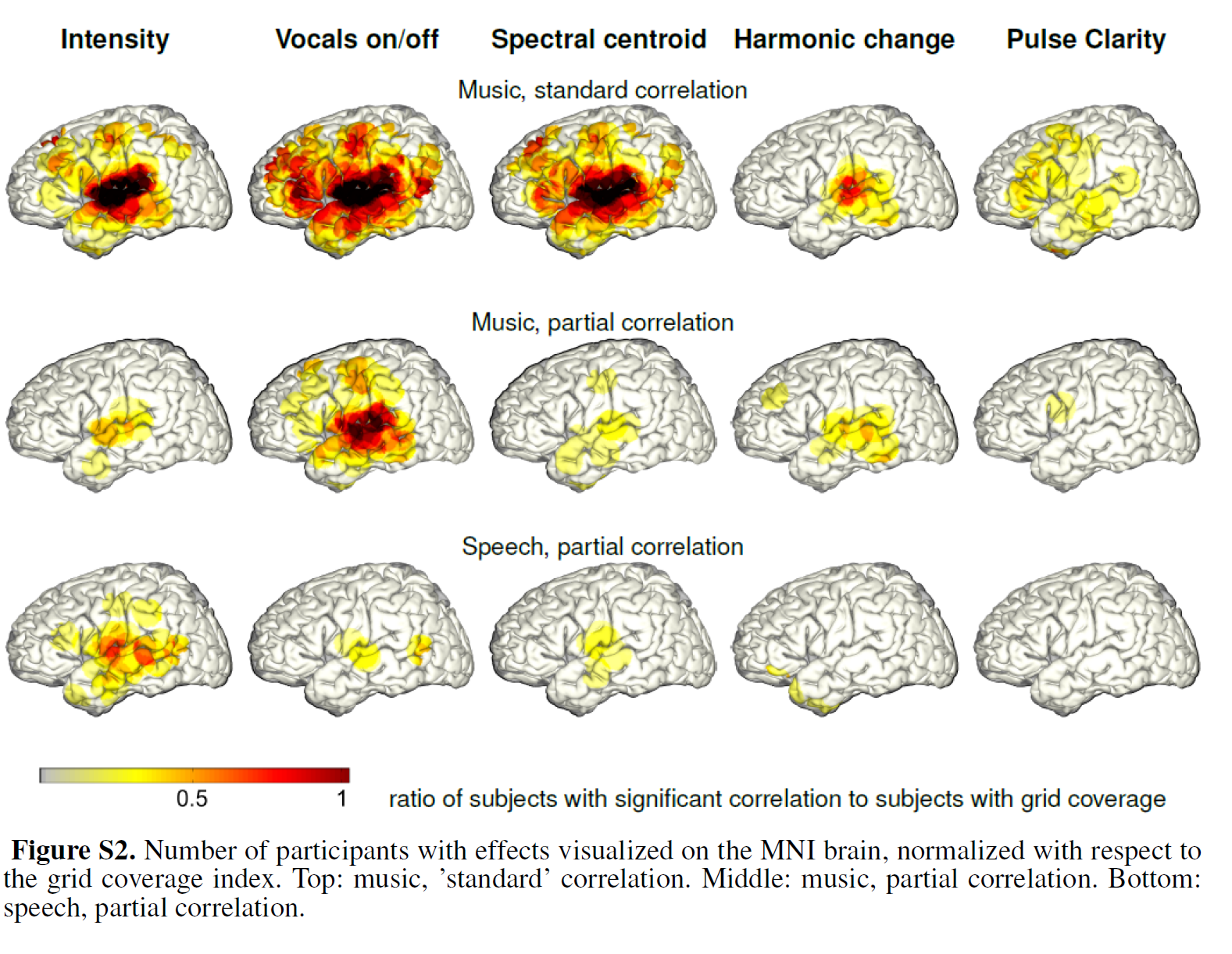

Supplement: Supplementary file 3 [file Image2.TIF]

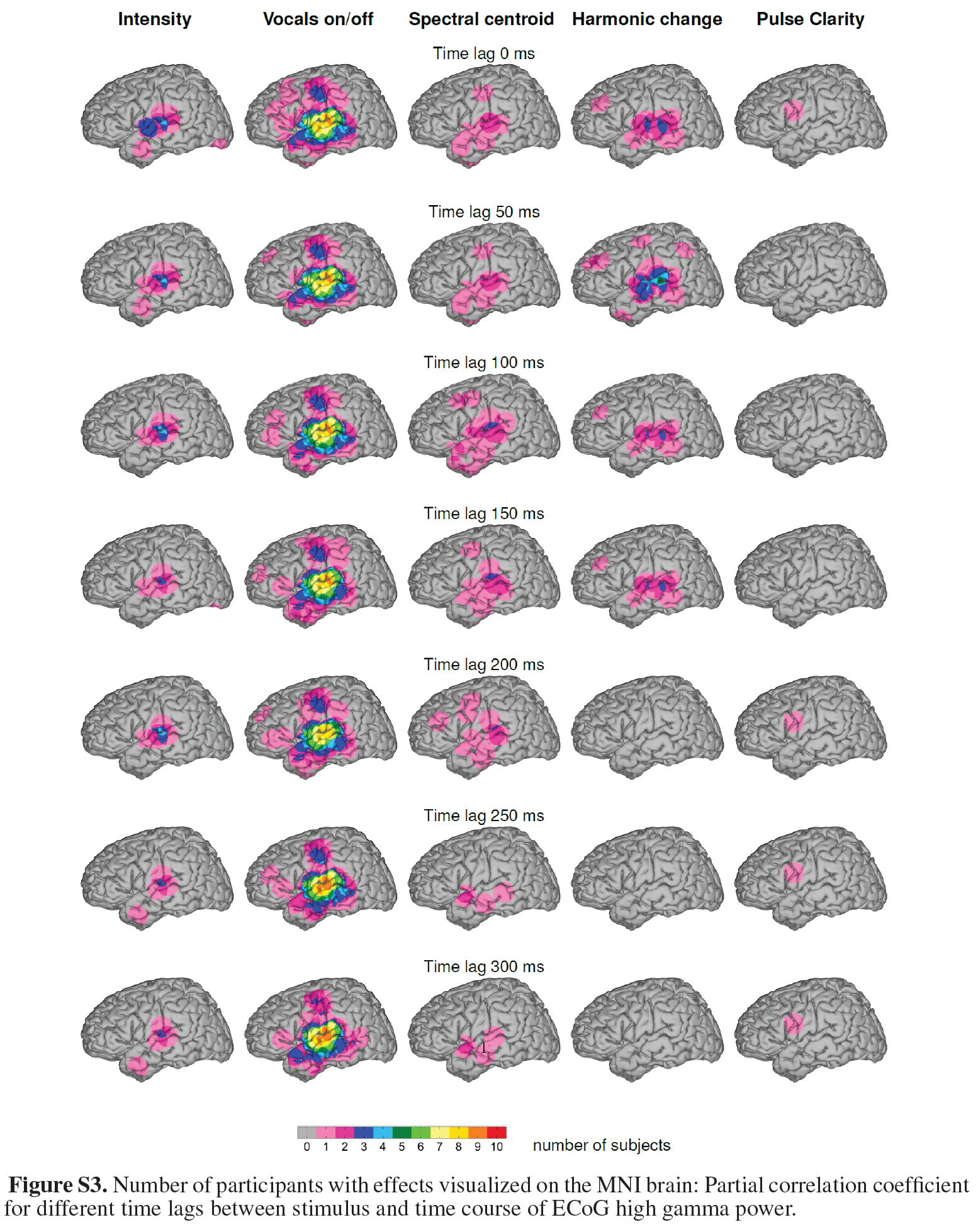

Supplement: Supplementary file 4 [file Image3.TIF]
